# A Protective Role of Aryl Hydrocarbon Receptor Repressor in Inflammation and Tumor Growth

**DOI:** 10.3390/cancers11050589

**Published:** 2019-04-27

**Authors:** Christoph F. A. Vogel, Yasuhiro Ishihara, Claire E. Campbell, Sarah Y. Kado, Aimy Nguyen-Chi, Colleen Sweeney, Marius Pollet, Thomas Haarmann-Stemmann, Joseph M. Tuscano

**Affiliations:** 1 Department of Environmental Toxicology, University of California, One Shields Avenue, Davis, CA 95616, USA; 2 Center for Health and the Environment, University of California, One Shields Avenue, Davis, CA 95616, USA; yaishihara@ucdavis.edu (Y.I.); cecampbell@ucdavis.edu (C.E.C.); sykado@ucdavis.edu (S.Y.K.); aimy.nguyenchi@gmail.com (A.N.-C.); mpollet@ucdavis.edu (M.P.); 3 Graduate School of Integrated Sciences for Life, Hiroshima University, Hiroshima 739-8521, Japan; 4 Department of Biochemistry & Molecular Medicine, School of Medicine, University of California, Davis, CA 95817, USA; casweeney@ucdavis.edu; 5 Leibniz Research Institute for Environmental Medicine, 40225 Düsseldorf, Germany; haarmann@uni-duesseldorf.de; 6 Division of Hematology & Oncology, Department of Internal Medicine, University of California Davis Comprehensive Cancer Center, Sacramento, CA 95817, USA; jtuscano@UCDAVIS.EDU

**Keywords:** AhR, AhRR, carcinogenicity, C/EBPβ, cyclooxygenase 2, inflammation, interleukin 1, lymphoma, TCDD

## Abstract

The aryl hydrocarbon receptor (AhR) is known for mediating the toxicity of environmental pollutants such as dioxins and numerous dioxin-like compounds, and is associated with the promotion of various malignancies, including lymphoma. The aryl hydrocarbon receptor repressor (AhRR), a ligand-independent, transcriptionally inactive AhR-like protein is known to repress AhR signaling through its ability to compete with the AhR for dimerization with the AhR nuclear translocator (ARNT). While AhRR effectively blocks AhR signaling, several aspects of the mechanism of AhRR’s functions are poorly understood, including suppression of inflammatory responses and its putative role as a tumor suppressor. In a transgenic mouse that overexpresses AhRR (AhRR Tg) we discovered that these mice suppress 2,3,7,8-tetrachlorodibenzo-p-dioxin (TCDD)- and inflammation-induced tumor growth after subcutaneous challenge of EL4 lymphoma cells. Using mouse embryonic fibroblasts (MEF) we found that AhRR overexpression suppresses the AhR-mediated anti-apoptotic response. The AhRR-mediated inhibition of apoptotic resistance was associated with a suppressed expression of interleukin (IL)-1β and cyclooxygenase (COX)-2, which was dependent on activation of protein kinase A (PKA) and the CAAT-enhancer-binding protein beta (C/EBPβ). These results provide mechanistic insights into the role of the AhRR to suppress inflammation and highlight the AhRR as a potential therapeutic target to suppress tumor growth.

## 1. Introduction

Human exposure to dioxins and dioxin-like compounds has been associated with a range of toxic effects including human carcinogenicity at multiple sites in occupational settings. Numerous dioxin-like compounds (polybrominated dibenzo-p-dioxins, dibenzofurans, biphenyls, and polycyclic aromatic hydrocarbons) can be abundant in the environment and have the potential to contribute to our daily background exposure. It has been previously shown that they mediate their toxicity through activation of the aryl hydrocarbon receptor (AhR) [1]. 2,3,7,8-tetrachlorodibenzo-p-dioxin (TCDD) as the prototypical ligand of AhR acts as a potent tumor promoter in various animal models and may cause tumors at multiple sites [2]. Exposure to dioxin and dioxin-like compounds has been shown to be carcinogenic and especially associated with the development of non-Hodgkin lymphoma (NHL) in mice [3,4] and in humans [5]. A recent meta-analysis demonstrated that the degree of external exposure and the blood levels of TCDD are significantly associated with cancer incidence and mortality, especially for NHL [6]. The AhR may also act as a critical receptor protein that mediates carcinogenesis independent of exogenous ligands. AhR activation via endogenous ligands can represent a critical event in human carcinogenesis and can result in the development of immune tolerance and increased survival of cancer cells [7]. The mechanisms of TCDD-mediated toxicity and AhR-dependent tumor promoting activity have been investigated in numerous studies with a focus on TCDD’s action as a potent hepatic tumor promoter [2,8]. On the other hand, the mechanism of the AhR Repressor (AhRR) acting as a tumor suppressor is poorly understood and has not been assessed in vivo.

AhRR, first described by Mimura et al. [9], has been identified as a bHLH-PAS protein but in contrast to AhR, the AhRR does not express a functional ligand-binding and transactivation domain. The AhRR has the ability to dimerize with the AhR nuclear translocator (ARNT) and act as a specific inhibitor of AhR activity through the competition of AhRR with AhR to form heterodimers with ARNT which hinders binding and transactivation of AhR/ARNT complexes via dioxin response elements (DREs). However results of transfection experiments with ARNT and AhRR mutants suggest a more complex mechanism in addition to the negative feedback mechanism through sequestration of ARNT and inhibition of the AhR signaling pathway [10,11]. Recent studies also indicate that repression of CYP1A1 activity is not always related to the expression level of AhRR [12]. These findings and a recent study showing the structural analysis of AhRR/ARNT [13], support that AhRR may interact with other transcription factors as proposed in the transrepression model by Evans et al. [14] and by our own study using a transgenic mouse model overexpressing AhRR [15].

The ability of AhRR to regulate AhR’s signaling in major cellular processes, such as cell cycling, inflammation, and apoptosis is complex and variable depending on the cellular and environmental context. In vitro experiments with several different types of cancer cell lines showed that the AhRR may act as a tumor suppressor gene [16,17]. Interestingly, a recent study identified numerous DNA binding sites in gene promoter regions of tumor suppressor genes and genes involved in carcinogenesis uniquely bound by AhRR [18]. In addition, epigenetic modifications of AhRR’s regulatory region have been found in human studies which was associated with the exposure to cigarette smoke and development of various types of cancer, e.g., [19]. Recently we established a transgenic mouse (AhRR Tg mice) that overexpresses AhRR and discovered that these mice were protected from TCDD-induced lethality that correlated with a reduction in inflammatory response and acute hepatotoxicity [15]. The current study was designed to test the tumor suppressive function of AhRR and to better understand the regulatory mechanisms of AhRR in inflammatory and cellular responses contributing to tumor promotion.

## 2. Results

### 2.1. Suppression of Tumor Growth in AhRR Tg Mice

**Figure 1:**
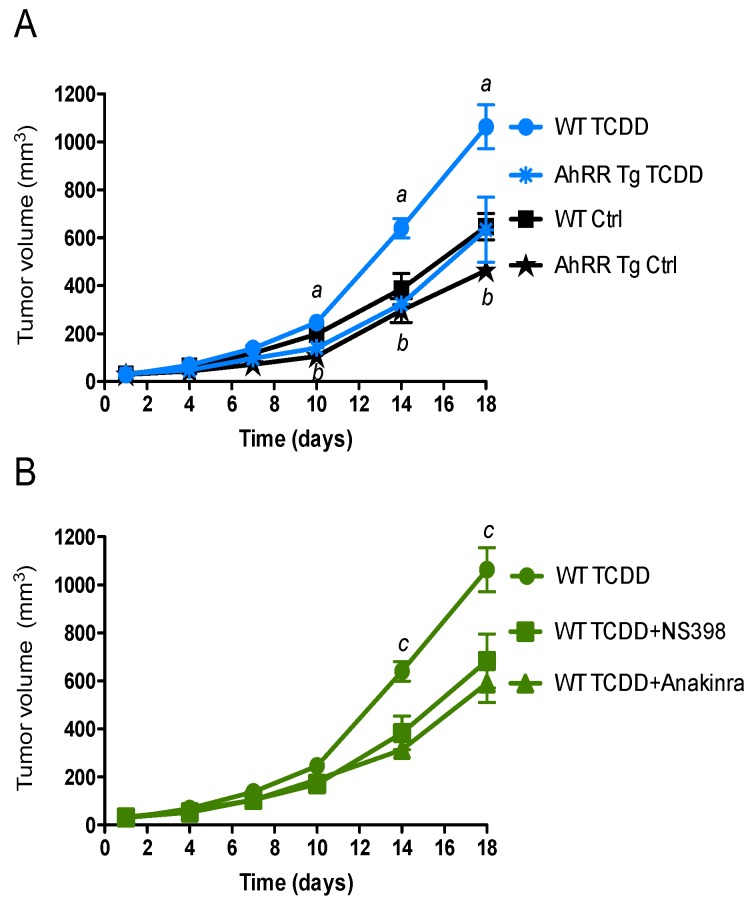
Suppressed tumor growth of EL4 lymphoma cells in AhRR Tg mice. Tumor volume of 10-week-old AhRR Tg mice and littermate WT mice (*n* = 6 for each group) following subcutaneous injection with EL4 lymphoma cells is shown. (**A**) After 24 h mice were treated with corn oil (Ctrl, black lines) or i.p. injected with 10 μg/kg TCDD (blue lines). (**B**) WT mice were treated with NS398 or Anakinra (green lines) 30 min. before and every 4 days after injection of TCDD. Tumor volume was measured over a period of 18 days. Means of tumor volume are shown. *^a^* significantly higher than AhRR Tg TCDD. *^b^* significantly lower than WT Ctrl. *^c^* significantly higher than mice treated with NS398 or Anakinra. (*p* < 0.01).

To address the tumor-suppressive action of AhRR in vivo, we used a syngeneic murine tumor model to assess changes in tumor susceptibility associated with inflammation. A common tumor model to define host resistance against transplanted tumors in vivo is the xenograft model with EL4 lymphoma cells. The results indicate a significantly suppressed tumor growth of EL4 lymphoma in AhRR Tg mice compared to wt B6 mice with or without TCDD treatment (Figure 1). Tumor growth of EL4 lymphoma was enhanced in TCDD-treated wt mice compared to control wt mice (Figure 1A). Furthermore, the TCDD-stimulated tumor growth of EL4 cells in wt mice was suppressed in the presence of the IL-1 receptor antagonist anakinra and NS-398, a selective COX-2 inhibitor (Figure 1B) supporting our hypothesis that TCDD in part mediates its tumor promoting activity through the IL-1 receptor and COX-2.

### 2.2. Resistance of AhRR Tg Mice to LPS

**Figure 2:**
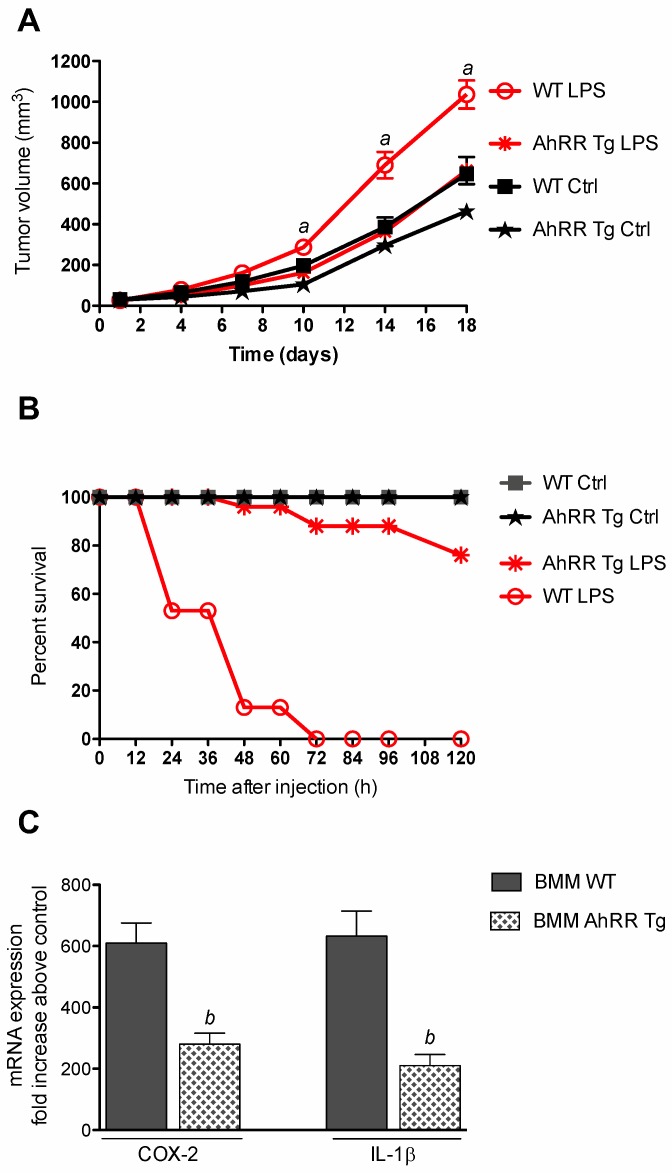
Resistance of AhRR Tg mice to LPS. (**A**) Suppressed growth of LPS-induced tumor volume of EL4 lymphoma cells in AhRR Tg mice. WT and AhRR Tg mice were treated i.p. with a single injection of LPS (2.5 mg/kg bw; red lines). Tumor volume was measured over a period of 18 days. Means of tumor volume are shown. *^a^* significantly higher than LPS-treated AhRR Tg and WT Ctrl mice (*p* < 0.01). (**B**) 6-week-old male AhRR Tg mice and littermate WT mice (*n* = 8 for each group) were i.p. injected with 25 mg/kg of LPS (red lines) or with the vehicle control PBS (black lines). Lethality was observed over 120 h after LPS challenge. (**C**) AhRR suppresses LPS-induced expression of IL-1β and COX-2 in BMM. BMM derived from WT and AhRR Tg mice were treated with 100 ng/mL LPS for 24 h and mRNA was analyzed by qPCR. *^b^* significantly lower than LPS-treated BMM WT (*p* < 0.005).

In order to test if AhRR suppresses inflammation-dependent tumor growth we treated mice with LPS after subcutaneous injection of EL4 lymphoma cells. The results indicate an enhanced tumor growth of EL4 lymphoma in LPS-treated wt mice compared to non-treated control wt mice (Figure 2A). LPS-stimulated tumor growth of EL4 cells was significantly reduced in AhRR Tg mice compared to wt mice. In order to further test the responsiveness of AhRR Tg mice toward LPS, we treated wt and AhRR Tg mice with a lethal dose of 25 mg/kg of LPS (Figure 2B). The lethality was observed over a period of 120 h after LPS injection. 50% of the wt mice died in the first 24 h after LPS treatment. At 48 h 90% of wt mice died after LPS and all wt mice were dead after 72 h of treatment. In contrast, none of the AhRR Tg mice died in the first 36 h of LPS treatment and 90% survived after 60 h of LPS treatment. Only 20% of the AhRR Tg mice died after LPS at the end of the observation period at 120 h. Furthermore, we used LPS-sensitive bone marrow-derived macrophages (BMM) to test if AhRR suppresses LPS-induced expression of IL-1β and COX-2 (Figure 2C). BMM derived from wt and AhRR Tg mice were treated with 25 ng/mL LPS for 24 h and mRNA was analyzed by qPCR. The results show a clear induction of COX-2 (610-fold) and IL-1β (633-fold) in BMM wt after LPS treatment. The LPS-induced expression of both, IL-1β and COX-2 was more than 50% repressed in BMM AhRR Tg.

### 2.3. AhRR Suppresses TCDD-Induced Expression of Inflammatory Markers

**Figure 3:**
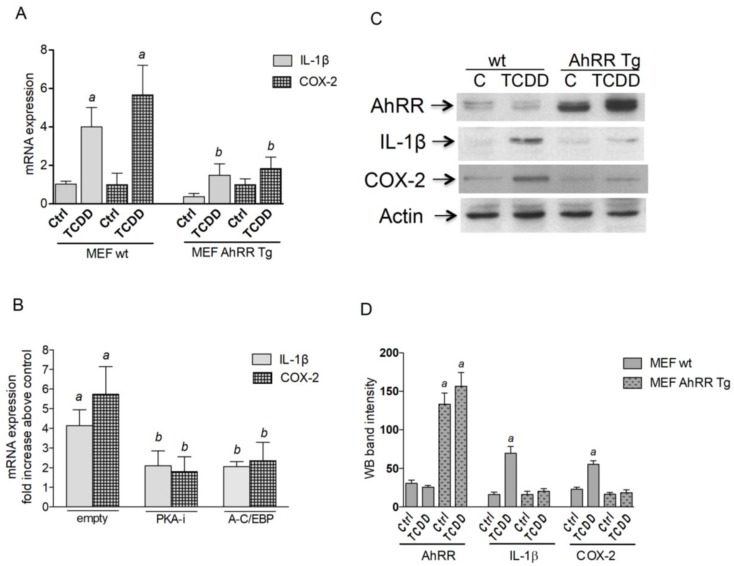
AhRR suppresses TCDD-induced expression of COX-2 and IL-1β in MEF. (**A**) MEF from wt and AhRR Tg mice were treated with 1 nM TCDD for 24 h and mRNA expression of COX-2 and IL-1β was analyzed by qPCR. *^a^* significantly higher than Ctrl; *^b^* significantly lower than wt, *p* < 0.05. (**B**) TCDD-induced expression of COX-2 and IL-1β is PKA and C/EBP dependent. MEF wt were transfected with an empty vector as control and dominant negative expression vectors PKA-i or A-C/EBP. After 16 h transfection MEF wt were treated with 1 nM TCDD for 24 h and mRNA expression was analyzed by qPCR. *^a^* significantly higher than Ctrl; *^b^* significantly lower than wt, *p* < 0.05. (**C**) Repressed protein level of IL-1β and COX-2 in AhRR Tg MEF determined by Western blot. MEF derived from wt or AhRR Tg mice were treated for 24 h with 1 nM TCDD or vehicle (0.1% DMSO). (**D**) densitometric evaluation of band intensities of the western blot bands of MEF wt (open bars) and MEF AhRR Tg (shaded bars) is presented. Results of three separate experiments are shown as mean values ± S.D. *^a^* significantly different from control cells (*p* < 0.05).

To test the role of AhRR in inflammatory responses in vitro we used mouse embryonic fibroblasts (MEF) from wt and AhRR Tg mice. MEF were treated with 1 nM TCDD for 24 h and RNA was extracted to analyze the expression of IL-1β and COX-2. The mRNA expression of both, IL-1β and COX-2 was induced in MEF wt by TCDD after 24 h of treatment (Figure 3A). The TCDD-induced expression of IL-1β and COX-2 mRNA was significantly repressed in MEF overexpressing AhRR derived from AhRR Tg mice compared to wt MEF. As reported earlier, the TCDD-mediated induction of COX-2 may involve the activation of PKA and DNA binding of C/EBPβ [20,21]. Therefore, we transiently transfected MEF wt with an empty vector as control and dominant negative expression vectors PKAi and A-C/EBP to inhibit PKA activation and DNA binding to C/EBP binding elements. The results indicate that inhibition of PKA or C/EBP binding significantly inhibits TCDD-induced expression of IL-1β as well as COX-2 (Figure 3B). MEF treated in Figure 3A were not transfected with “empty” vector but levels of IL-1β and COX-2 are not significantly different from TCDD-treated and MEF transfected with an empty vector in Figure 3B. Western blot analysis confirmed that the repressed mRNA expression in AhRR Tg MEF affects the protein level of IL-1β and COX-2 (Figure 3C). TCDD treatment increased the level of AhRR in AhRR Tg MEF but inhibited the increase of COX-2 and IL-1β. The effect of TCDD on AhRR protein level was not detected in wt MEF.

### 2.4. Repressed DNA-Binding Activity of C/EBP in AhRR Tg MEF

**Figure 4:**
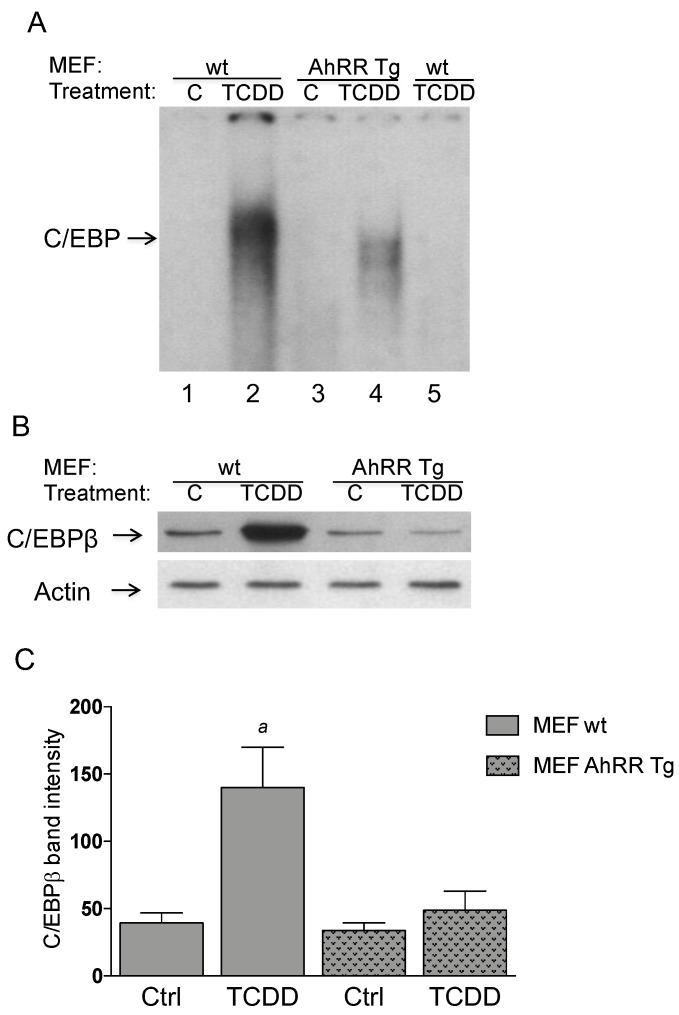
AhRR represses the levels and activity of C/EBP. (**A**) Repressed DNA binding activity to a C/EBP consensus element in AhRR Tg MEF compared to wt MEF. MEF from wt (lanes 1 and 2) and AhRR Tg mice (lanes 3 and 4) were treated with 1 nM TCDD (lanes 2 and 4). After 4 h nuclear proteins were extracted. For specificity a 200-fold molar excess of unlabeled probe was added as competitor (lane 5). (**B**) Repressed protein level of C/EBPβ in AhRR Tg MEF. MEF from wt and AhRR Tg mice were treated with 1 nM TCDD for 4h. Nuclear proteins were extracted and protein level of C/EBPβ was determined by western blot. (**C**) densitometric evaluation of band intensities of the western blot bands of MEF wt (open bars) and MEF AhRR Tg (shaded bars) is presented. Results of three separate experiments are shown as mean values ± S.D. *^a^* significantly different from control cells (*p* < 0.05).

The nuclear protein binding to the key responsive element C/EBP that is frequently found in the promoters of pro-inflammatory genes and known to mediate expression of IL-1β and COX-2 was assessed using electrophoretic mobility shift assay (EMSA). Previously we found that enhanced expression of AhRR in mice repressed TCDD-induced up-regulation and nuclear accumulation of C/EBPβ in vivo [15] and that the activity of C/EBPβ is important to mediate the induction of COX-2 by TCDD [20]. We confirmed our initial findings in MEF from wt and AhRR Tg mice which serve as a suitable in vitro model to study the enhanced expression of AhRR. The results show a repressed binding activity of C/EBP (Figure 4A) associated with suppressed protein levels of C/EBPβ (Figure 4B) in the nuclei of TCDD-treated MEF overexpressing AhRR.

### 2.5. TCDD-Induced PKA Activity is Reduced in AhRR Tg MEF

Previously we have shown that TCDD activates PKA, which mediates the activation of C/EBPβ [21]. MEF were treated with 1 nM TCDD and PKA activity was determined after 2 h. TCDD increased the basal PKA activity by 2.8-fold in MEF wt (Table 1). The TCDD-induced basal PKA activity was significantly diminished in MEF AhRR Tg compared to MEF wt; however, treatment with TCDD still increased PKA activity by 1.8-fold in MEF AhRR Tg compared to vehicle (0.1% DMSO) controls. The total PKA activity was elevated 1.4-fold by TCDD in MEF wt and was significantly decreased in MEF AhRR Tg. The specific AhR antagonists MNF and CH223191 completely suppressed the TCDD-induced PKA activity indicating that the AhR is required in this process. The total PKA activity is cofactor dependent and measured after addition of cAMP. The basal activity, a measure of active PKA at the time of harvest, is measured in the absence of exogenous cAMP. The basal PKA activity was on average less than 10% of the total PKA activity.

#### 2.5.1. PKA and C/EBPβ Mediate TCDD-Induced IL-1β Promoter Activity

**Figure 5:**
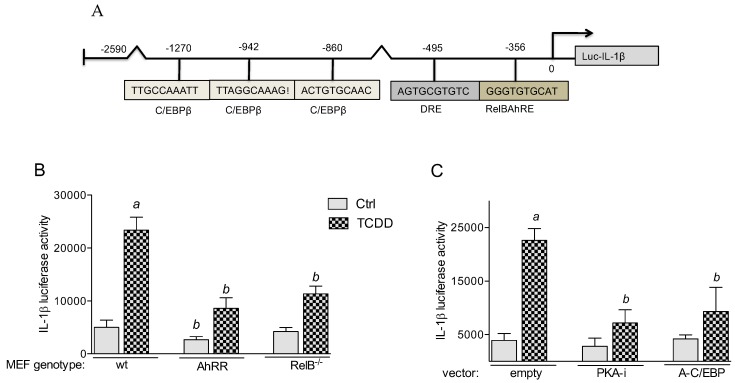
TCDD-induced IL-1β promoter activity is RelB and PKA-C/EBPβ-dependent. (**A**) Potential binding sites of the full-length promoter construct of the mouse *IL-1β* gene containing 2590 bp upstream of the transcriptional start site (indicated by an arrow) cloned into a luciferase (*luc*) reporter vector. Positions of one putative DRE consensus, one non-consensus DRE (RelBAhRE) and three recognition sites for C/EBPβ are presented. (**B**) MEF from wt, AhRR Tg, and RelB^−/−^ mice were transfected with a luciferase reporter construct containing 2590 bp (IL-1β) of the mouse gene promoter region. (**C**) MEF wt were cotransfected with an empty, PKA inhibitor (PKA-i) and C/EBP dominant negative expression plasmid. 24 h after transfection, MEF were treated with 1 nM TCDD for 6 h. Relative luciferase activity units are given as mean values of triplicates as a result of three independent experiments. *^a^* significantly different from control cells (*p* < 0.05); *^b^* significantly lower than MEF wt or cells co-transfected with empty vector (*p* < 0.05).

*IL-1β* was identified as an AhR target gene [22] and as a prototypical gene suppressed by AhRR [15,23]. Therefore, we selected the mouse *IL-1β* gene promoter to identify potential C/EBPβ and AhR binding sites (canonical and non-canonical DREs), which could be affected by AhRR. Using the TFSEARCH program we identified a consensus DRE site and a non-canonical AhR/RelB binding element (RelBAhRE) for mouse *IL-1β* (Figure 5A). Three potential binding elements for C/EBPβ were identified on the promoter region of the mouse *IL-1β* gene. A luciferase reporter construct has been generated for mouse *IL-1β*, which is inducible by TCDD. The results show that TCDD-induced IL-1β activity is significantly repressed in MEF derived from AhRR Tg and from RelB^−/−^ mice (Figure 5B). Co-transfection studies with a PKA inhibitor (PKA-i) and C/EBP dominant negative expression plasmid (A-C/EBP) significantly blocked the TCDD-mediated activation of IL-1β promoter activity by about 50% (Figure 5C). The results indicate the importance of RelB and PKA/C/EBPβ in mediating the transcriptional activation of the *IL-1β* gene by TCDD. Promoter analyses of COX-2 and IL-8 in our previous studies confirm the important role of PKA, C/EBPβ, and RelB in TCDD-mediated gene induction [20,21,24].

#### 2.5.2. Enhanced Recruitment of AhR to a RelBAhRE Binding Site of the IL-1β Promoter

**Figure 6:**
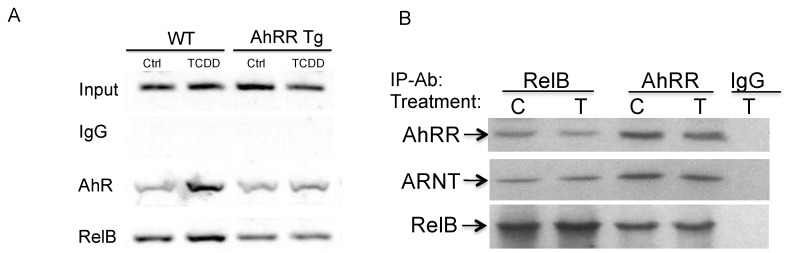
TCDD stimulates the recruitment of AhR and RelB to the RelBAhRE region of the mouse *IL-1β* promoter. (**A**) MEF derived from wt and AhRR Tg mice were treated with 1 nM TCDD for 90 min. ChIP assays were performed using AhR- and RelB-specific antibodies followed by PCR analysis with primer pairs covering the specified RelBAhRE region of mouse *IL-1β* promoter. (**B**) Association of AhRR with ARNT and RelB. Co-IP of AhRR, ARNT, and RelB with RelB and AhRR antibody. MEF from wt and AhRR Tg mice were treated with 1 nM TCDD (T) or DMSO (C) for 90 min and cell lysates were used for co-IP. Western blot analysis was performed to detect specific association of AhRR with ARNT and RelB. Rabbit lgG was used as negative control.

ChIP assays with antibodies against AhR and RelB proteins were analyzed by PCR using primer pairs covering the specified RelBAhRE region of mouse *IL-1β* (Figure 6A). Genomic DNA and the sonicated input DNA were separated by agarose gel electrophoresis and visualized by ethidium bromide staining. ChIP assay samples from wt MEF and AhRR Tg MEF were analyzed as described [24]. The results show that TCDD stimulates the recruitment of AhR to the RelBAhRE site of *IL-1β* 90 min. after treatment (Figure 6A). The results indicate that AhRR inhibits the recruitment of TCDD-activated AhR and RelB to the potential RelBAhRE site of the mouse *IL-1β* gene.

#### 2.5.3. Physical Association of AhRR, ARNT, and RelB

Here we tested the hypothesis that the AhRR in complex with ARNT interacts with RelB. The AhRR can form heterodimers with ARNT to repress AhR-dependent transactivation [14]. Moreover, it was shown that ARNT interacts with RelB [25] resulting in inhibition of RelB-dependent apoptosis [26]. The physical association between AhRR, ARNT and RelB was analyzed in co-immunoprecipitation (co-IP) studies. Results indicate the interaction of AhRR not only with ARNT but also with RelB in absence or presence of an AhR ligand (Figure 6B). TCDD did not affect the apparent association between AhRR, ARNT, and RelB.

### 2.6. Inhibition of TCDD-Induced Apoptotic Resistance by AhRR

**Figure 7:**
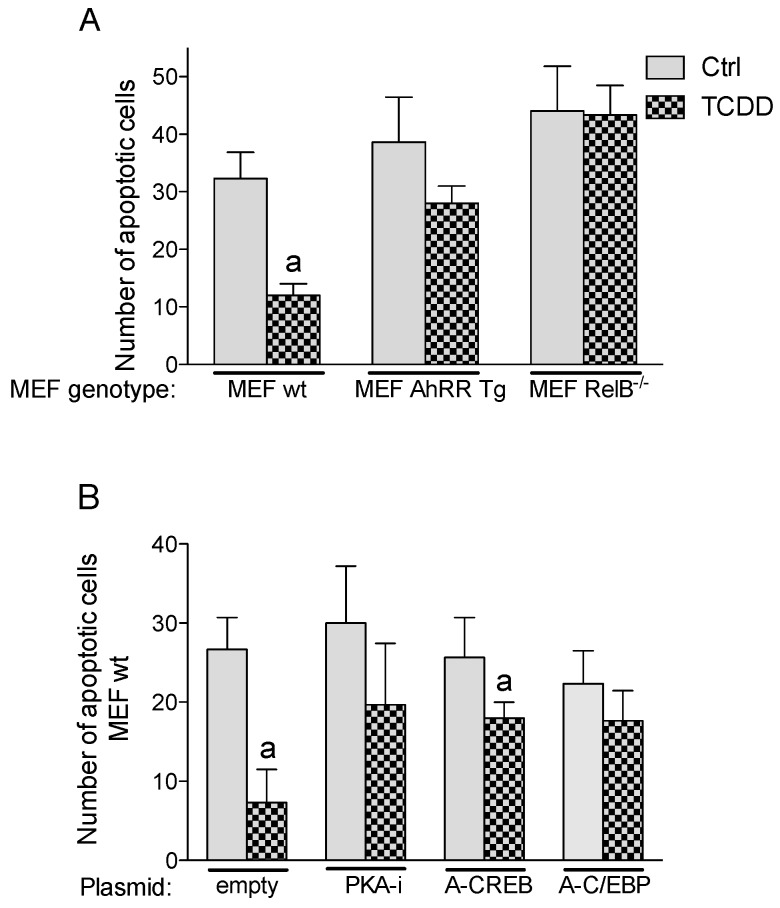
TCDD-mediated apoptotic resistance in MEF. (**A**) MEF derived from wt, AhRR Tg, and RelB^−/−^ mice were treated with 1 nM TCDD for 24 h. Apoptosis was induced by UV light and the number of apoptotic cells was counted after 4 h. (**B**) MEF wt were transfected with dominant negative expression vectors PKA-i, A-CREB and A-C/EBP for 16 h followed by treatment with 1 nM TCDD for 24 h. Apoptosis was induced by UV light and the number of apoptotic cells was counted after 4 h. Values are averages of duplicates from three independent experiments. ^a^ Significantly lower than control *p* < 0.05.

Apoptosis is a programmed cell death to eliminate dysfunctional or damaged cells. The ability to escape apoptosis is an important characteristic of malignant cells and the development of cancer. Therefore, we tested how AhRR affects the apoptotic response. A standard UV-induced apoptosis test using the Annexin V-FITC Apoptosis Detection Kit (Sigma) was applied as described [4,27]. MEF isolated from wt and AhRR Tg mice were used to examine the enhanced expression of AhRR on TCDD-mediated apoptotic resistance. The exposure to UV light induced apoptosis in wt and AhRR Tg MEF (Figure 7). MEF derived from wt mice showed that activation of AhR by TCDD has a significant inhibitory effect on UV-induced apoptosis. AhRR overexpression inhibited TCDD-mediated apoptotic resistance (Figure 7A). Furthermore, our results show that inhibition of PKA and CREB (required for activity of C/EBPβ) as well as C/EBP DNA binding hinders TCDD’s anti-apoptotic response in MEF wt (Figure 7B).

## 3. Discussion

The results of the current study show that transgenic mice overexpressing AhRR inhibited EL4 lymphoma growth in presence or absence of the exogenous AhR ligand TCDD. Furthermore, inflammatory conditions induced by LPS stimulated tumor growth of EL4 lymphoma cells in wt mice, which was also suppressed in AhRR Tg mice indicating that AhRR may suppress tumor growth independent of exogenous and toxic AhR ligands. Interestingly, the AhR has been shown to act as a critical receptor protein that mediates tumor development independent of exogenous ligands [7]. The authors concluded that the continuing activation of AhR via endogenous ligands such as kynurenine represent a critical event in tumor promotion resulting in increased survival of cancer cells. Thus the TCDD-independent yet AhRR-mediated tumor suppression is an important aspect, which may depend on endogenous ligands produced by the tumor microenvironment or tumor cells directly.

Recently we reported that the most consistently observed trend found in TCDD-treated AhRR Tg mice is an overall reduction in the responsiveness of several inflammation markers, especially IL-1β and COX-2 [15]. The current study confirmed the repressed induction of IL-1β and COX-2 in MEF and BMM overexpressing AhRR. Both inflammatory genes, COX-2 and IL-1β play a critical role in carcinogenesis. We have previously shown, that C/EBPβ and COX-2 are important mediators of an AhR-dependent and TCDD-induced resistance to an apoptotic response in lymphoma cells demonstrating the critical role of COX-2 in the pathogenesis of lymphoma [4]. A previous report and own data underlined the importance of C/EBPβ and COX-2 in the pathogenesis of lymphoma [28,29]. COX-2 is an inducible isoform upregulated in many cancers [30] and selective inhibition of COX-2 has been shown to significantly increase apoptosis in tumors [31]. Degner et al. [32] have shown that AhR ligands can upregulate COX-2 expression, which led to a pro-inflammatory local environment that supported tumor development.

Furthermore, our results suggest that IL-1R signaling is an important mediator of TCDD-stimulated growth of EL4 lymphoma cells in vivo. An interesting study using “triple-null” mice lacking IL-1 and TNFα receptors was performed in Chris Bradfield’s laboratory and demonstrated the important role of IL-1β mediating TCDD’s toxic effects such as liver inflammation and tumor promoting effects [33,34]. Early processes of carcinogenesis have been shown to be promoted via IL-1β signaling and the pro-inflammatory microenvironment [35]. Furthermore, dysregulated levels of inflammatory mediators are likely to play a key role in carcinogenesis mediated via environmental exposure as recently reviewed [36]. The critical role of IL-1β was also demonstrated in inflammation-driven carcinogenesis models using IL-1β-deficient mice treated with the procarcinogen and AhR ligand 3-methylcholanthrene (3-MC) [37]. Furthermore, transgenic mice that overexpress IL-1β developed gastric inflammation and gastric cancer [38], a response also found in transgenic mice expressing a constitutively active AhR [39]. Noticeably, we observed suppressed expression of COX-2 and IL-1β associated with a reduced nuclear accumulation of C/EBPβ in AhRR Tg mice [15] and AhRR Tg MEF. AhRR inhibits TCDD-mediated activation of PKA, which has been shown to induce DNA binding activity of C/EBPβ [21]. Besides putative AhR binding sites, DNA binding elements for C/EBPβ contribute to transcriptional activation of IL-1β and COX-2 [40,41]. We previously demonstrated that PKA-mediated activation of C/EBPβ is a key transactivator for AhR-mediated *Cox-2* gene induction [20]. Interestingly, studies have shown that PKA is critically involved in ligand independent activation of AhR and the non-canonical AhR signaling pathway [24,42]. The current results show that TCDD-induced IL-1β expression is also repressed when PKA and DNA binding of C/EBP are blocked. Furthermore, we identified a RelB/AhR binding element on the promoter of IL-1β and found RelB to be important mediating the expression of IL-1β confirming a previous study in dendritic cells [43]. Additionally, we found that AhRR represses the induction of IL-1β and COX-2 in macrophages after stimulation with LPS. Furthermore, AhRR Tg mice were protected from LPS shock as well as LPS-induced tumor growth, which presents another important tumor suppressing function of AhRR since inflammatory processes are major contributing factors promoting cancer development [44].

Activation of cAMP-dependent PKA as part of the non-canonical AhR pathway, leads to protein phosphorylation including the cAMP response element binding protein (CREB), which regulates activity of C/EBPβ [45]. C/EBPβ has been found to be an important factor in the autocrine survival pathway of myeloid tumor cells [46]. Hyperproliferation and transformation of the normal mammary epithelial cells such as MCF10A cells has been found to be mediated by increased expression of C/EBPβ [47]. On the other hand, deficiency of C/EBPβ led to an increased (17-fold) apoptosis of epidermal keratinocytes in mice after treatment with a carcinogen. Furthermore, C/EBPβ null mice were completely resistant to carcinogen-induced skin tumorigenesis and seem to be protected against lymphomas in a carcinogenesis model compared to wt mice [48]. Inhibition of C/EBPβ in transgenic mice caused regression of papillomas with an associated increase in apoptosis [49]. Previously we have shown that TCDD inhibits UV-induced apoptosis in lymphoma cells, which requires functional expression of COX-2 and C/EBPβ [4]. Furthermore, current data show that TCDD’s anti-apoptotic cell response is significantly reduced in MEF from AhRR Tg mice, which requires PKA and C/EBP binding and which is completely abrogated in MEF RelB^−/−^. The results confirm data with ectopic expression of AhRR in human breast cancer cells [50] and previous reports showing that AhRR can act as a tumor suppressor against several types of cancers [16,17].

## 4. Materials and Methods

### 4.1. Reagents and Antibodies

Dimethyl sulfoxide (DMSO) was purchased from Sigma. [γ-^32^P]ATP (6000 Ci/mmol) was provided by ICN Biochemicals, Inc. (Costa Mesa, CA, USA). LPS isolated from *Escherichia coli* strain 055:B5 was purchased from (Sigma Aldrich, St. Louis, MO, USA). TCDD (>99% purity) was originally obtained from Dow Chemical Co. (Midland, MI, USA). Other molecular biological reagents were purchased from Cayman Chemicals (Ann Arbor, MI, USA) and Applied Biosystems (Foster City, CA, USA). AhRR Tg mice were genotype using the DNA/RNA Shield reagent (Zymo Research, Irvine, CA, USA) for nucleic acids isolation. The antibodies against actin (sc-1616), C/EBPβ (sc-150), IL-1β (sc-7884), and ARNT (sc-17811) were purchased from Santa Cruz Biotechnology (Santa Cruz, CA, USA). The purified rabbit anti-AhRR antibody was purchased from Novoprotein (Summit, NJ, USA), COX-2-specific polyclonal antibody and FICZ from Cayman Chemicals, AhR-specific polyclonal antibody from Enzo Life Sciences (Farmingdale, NY, USA) and RelB-specific polyclonal antibody from Active Motif (Carlsbad, CA, USA).

### 4.2. Cell Culture and Transfection Experiments

MEF were isolated from wild type and AhRR Tg B6 mouse embryos and were cultured in DMEM:F12 culture medium as described [51]. MEF were transiently transfected using jetPEI (PolyTransfection; Qbiogene, Irvine, CA, USA), according to the manufacturer's instructions. The transfection was allowed to proceed for 16 h, and cells were treated with 1 nM TCDD or 0.1% DMSO (control) for 24 h before UV-irradiation. The luciferase reporter construct containing the IL-1β promoter sequence was provided by SwitchGear Genomics (Menlo Park, CA, USA) corresponding to a −2590 bp of the mouse promoter sequence. The protein kinase A inhibitor expression vector (PKA-i) was kindly provided by Albert Smolenski (UCD Conway, Dublin, Ireland) and has been shown to reduce cAMP-dependent protein kinase activity, but not protein kinase C activity. The A-CREB and A-C/EBP vectors were kindly provided by Charles Vinson (NCI, Bethesda, MD, USA) and produce dominant-negative proteins that specifically inhibit CREB phosphorylation and the DNA binding of the C/EBP members, respectively.

### 4.3. Mice and Treatment

Female and male C57BL/6J wild type (wt) and AhRR Tg mice were housed in a selective pathogen-free facility at UC Davis. Mice were maintained on a 12:12 h light/dark cycle and had free access to water and food according to the guidelines set by the University of California. The protocol for animal care and use was approved and completed by the Institutional Animal Care and Use Committee (IACUC) on 10 December 2018 at the University of California Davis (#19671). This project was conducted in accordance with the ILAR guide for the care and use of laboratory animals, and the UC Davis Animal Welfare Assurance on file with the US Public Health Service. TCDD and LPS was administered via intraperitoneal (i.p.) injection for RNA, protein expression analysis, and LPS shock experiments. To address the tumor-suppressive action of AhRR in vivo, we used a syngeneic murine tumor model to evaluate in tumor susceptibility in wt and AhRR Tg mice. To create tumors, we used an orthotopic xenograft tumor model by subcutaneous (s.c.) injection of EL4 mouse lymphoma cells. The EL4 mouse lymphoma cells were established from a lymphoma induced in a B6 mouse by 9,10-dimethyl-1,2-benzanthracene [52]. The cultured EL4 tumor cell suspension was resuspended in PBS to obtain the desired EL4 tumor cell concentration of 500,000 cells/mL. A 1 mL syringe affixed with a 23-G needle was loaded with 0.1 mL of the EL4 tumor cell suspension (50,000 cells). For control, 100 mL PBS alone was injected. EL4 cells were injected subcutaneously into the right rear thigh of wt and AhRR Tg mice (10 weeks old, female and male with six animals in each group). 24 h after injection of EL4 cells, mice were treated with vehicle (corn oil or PBS) and TCDD (10 μg/kg bw) or LPS (2.5 mg/kg bw) in order to test possible enhancing effects of TCDD and the inflammatory stimulus LPS on tumor growth of EL4 lymphoma cells in wt and AhRR Tg mice. To block the effect of increased IL-1β production the IL-1 receptor antagonist anakinra was administered at 30 mg/kg. The COX-2 selective inhibitor NS-398 was administered at a dose of 36 mg/kg bw. Anakinra and NS-398 were administered i.p. 30 min before injection of TCDD. As a maintenance dose the inhibitors were administered at the original dose every 4 days according to its half-life in vivo. Doses and timing of anakinra and NS-398 were chosen based on their effectiveness in preventing disease endpoints in vivo [53,54]. Each mouse was palpated daily at the injection site and the tumor size was measured daily using a slide microcaliper for 24 days post-injection. These data were used to determine the tumor volume by employing the following formula V = (L×W×H)/2.

### 4.4. Electrophoretic Mobility Shift Assay (EMSA)

Nuclear extracts were isolated from MEF as described previously [24]. Cells were treated with LPS or TCDD for 90 min and harvested in ice cold Dulbecco’s PBS. The DNA/protein binding reactions were carried out in a total volume of 15 μL containing 10 μg of nuclear protein, 60,000 cpm of double-stranded C/EBP consensus oligonucleotide (5′-TGCAGATTGCGCAATCTGCA-3′) plus 1 μg of poly(dI·dC). The samples were incubated at room temperature for 20 min. Competition experiments were performed in the presence of a 100-fold molar excess of unlabeled oligo. Protein-DNA complexes were resolved on a nondenaturating polyacrylamide gel and visualized by exposure of the dried gels to x-ray films. Protein-DNA complexes were quantified using a ChemiImager^TM^ 4400 (Alpha Innotech Corp., San Leandro, CA, USA).

### 4.5. RNA Isolation and Real-Time PCR

Total RNA was isolated from cells using a Quick-RNA Mini prep isolation kit (Zymo Research), and cDNA synthesis was performed as described [4] using a cDNA synthesis kit Applied Biosystems (Foster City, CA, USA). Detection of β-actin and differentially expressed target genes was performed with a LightCycler LC480 Instrument (Roche Diagnostics, Indianapolis, IN, USA) using the Fast SYBR Green Master Mix (Applied Biosystems) according to the manufacturer's instructions. The primers for each gene were designed on the basis of the respective cDNA or mRNA sequences using OLIGO primer analysis software provided by Steve Rozen and the Whitehead Institute/Massachusetts Institute of Technology Center for Genome Research so that the targets were 100–200 bp in length. PCR amplification was carried as described [4]. To confirm the amplification specificity, the PCR products were subjected to melting curve analysis.

### 4.6. Protein Kinase A (PKA) Assays

PKA activity was determined in cell lysates using a PKA assay kit (Upstate Biotechnology Inc., Lake Placid, NY, USA) as described previously [21]. Briefly, the total PKA activity was measured by the addition of 2 μM cAMP; basal activity, a measure of active PKA at the time of harvest, was measured in the absence of exogenous cAMP. The amount of ^32^P was quantified by scintillation counting. Nonspecific activity was subtracted by using a PKA inhibitor peptide. To examine AhR-dependent effects, TCDD-treated cells were simultaneously treated with the AhR antagonists MNF (5 μM) or CH223191 (10 μM).

### 4.7. ChIP Assay

MEF cells were treated with TCDD for the indicated times and protein-DNA complexes were cross-linked with 1% formaldehyde for 10 min and prepared for ChIP assay as described [24]. DNA was purified using a DNA purification kit (Zymo Research) and eluted in 50 μL. ChIP DNA (5 μL) was amplified by real-time PCR with primers 5′-ATCCAGTTACCAAACTCCAAC-3′ and 5′-ATTGACACCATCTGCACAATT-3′ covering the specified region RelBAhRE of IL-1β to amplify a 188 bp fragment of the IL-1β promoter.

### 4.8. Nuclear Complex Co-Immunoprecipitation Assay

Preparation of nuclear extracts and co-immunoprecipitation was performed as described [21]. To analyze level of AhR and RelB protein in nuclei, nuclear protein extracts (15 μg) were separated on a 10% SDS-polyacrylamide gel and blotted onto a PVDF membrane (Immuno-Blot, BioRad, Hercules, CA, USA). The antigen-antibody complexes were visualized using the chemoluminescence substrate SuperSignal^®^, West Pico (Pierce, Rockford, IL, USA).

### 4.9. Western Blot Analysis

Whole cell lysates were prepared on ice with RIPA buffer containing protease inhibitor cocktail (Roche) as described [21]. The lysates were centrifuged at 16,000× *g* at 4 °C for 10 min, and the supernatants were collected as whole cell lysates. Whole cell lysates were separated on a SDS-polyacrylamide gel and blotted onto a PVDF membrane (Immuno-Blot, Bio-Rad). The antigen-antibody complexes were visualized using the chemiluminescence substrate SuperSignal^®^, West Pico (Pierce). For quantitative analysis, respective bands were quantified using a ChemilmagerTM 4400 (Alpha Innotech Corporation, San Leandro, CA, USA).

### 4.10. Apoptosis Assay on UV-Irradiated Cells

MEF cells (5 × 10^5^ cells) were seeded in a 6 cm dish and exposed to TCDD for 24 h prior to UV-irradiation and apoptosis was detected by Annexin V staining as described previously [4].

### 4.11. Statistical Analysis

All experiments were repeated a minimum of three times, and data are expressed as mean ± S.D. Differences were considered significant at *p* < 0.05. A comparison of two groups was made with an unpaired, two-tailed Student’s *t* test. A comparison of multiple groups was made with analysis of variance followed by a Dunnett’s or Tukey’s test.

## 5. Conclusions

**Figure 8:**
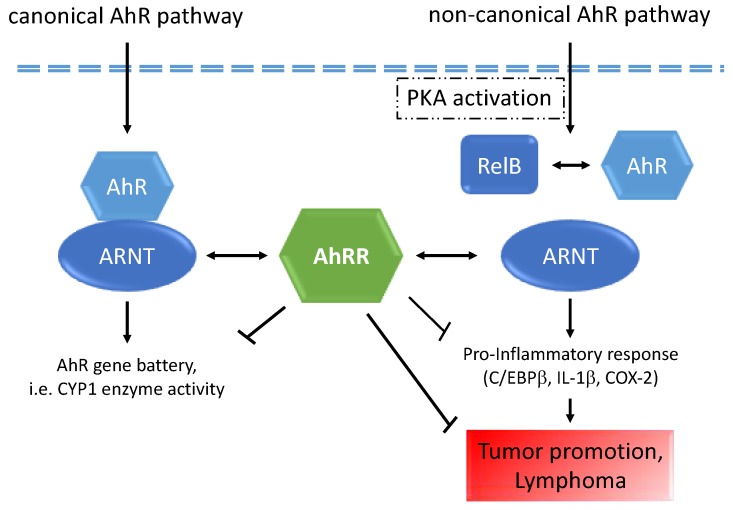
A schematic diagram illustrates our hypothesis how AhRR may interact with the canonical and non-canonical AhR signaling pathways as described recently [11]. AhRR may suppress inflammatory responses and tumor promotion via interaction with RelB in complex with AhR and ARNT. Double arrows depict the interaction of AhRR with ARNT and RelB with AhR.

In summary, our data indicate that the tumor suppressive function of AhRR is mediated via its interaction with the non-canonical AhR pathway (Figure 8) resulting in down-regulation of cellular inflammation through inhibition of the PKA-C/EBPβ inflammatory axis and inhibition of tumor growth and lymphoma.

## Figures and Tables

**Table 1:** Effect of AhRR on TCDD-mediated PKA activity.

Treatment	PKA Activity (pmol/min/mg Protein)			
	MEF wt		MEF AhRR Tg	
	basal	total	basal	total
Control	110 ± 20	2644 ± 150	95 ± 14	2450 ± 32
TCDD	290 ± 38 *^a^*	3720 ± 240 *^a^*	170 ± 25 *^b^*	2865 ± 80 *^b^*
MNF+TCDD	145 ± 28 *^c^*	2830 ± 110 *^c^*	126 ± 40 *^c^*	2050 ± 90 *^c^*
CH223191+TCDD	123 ± 21 *^c^*	2540 ± 140 *^c^*	102 ± 20 *^c^*	1980 ± 110 *^c^*

MEF cells from wt and AhRR Tg mice were exposed to 1 nM TCDD in presence or absence of the AhR antagonists MNF or CH223191. Total and basal PKA activities were measured after 2 h. The assay background has been subtracted by using a PKA inhibitor peptide. Values are the mean ± S.D. of triplicates and are *^a^* significantly higher than wt control (*p* < 0.005); *^b^* significantly lower than TCDD-treated wt cells (*p* < 0.005); *^c^* significantly lower than TCDD-treated wt and AhRR Tg cells (*p* < 0.005).
